# Unprofessional Conduct in a Member of the U. S. V. M. A.

**Published:** 1897-02

**Authors:** 


One of Pennsylvania's well-known veterinarians, a member of the United States Veterinary Medical Association, is surely paving the way for a summary invitation before the executive committee to answer charges of unprofessional conduct and violation of the code of ethics.

